# Supplementary material for: The Influence of Initial Immunosuppression on the Kinetics of Humoral Response after SARS-CoV-2 Vaccination in Patients Undergoing Kidney Transplantation
Source: Vaccines (Basel). 2024 Oct 3;12(10):1135. doi: 10.3390/vaccines12101135 (PMC11510881; doi:10.3390/vaccines12101135)
Supplement: Supplementary file 1 [file vaccines-12-01135-s001.zip › vaccines-3219551-supplementary.pdf]

Supplemental Material

Table S1. Donor demographic characteristics.

	**Transplant N = 113**
**Age**, years (IQR)	50 (34-59)
**Male sex**, N (%)	62 (54.9)
**Race**, N (%)	
White	60 (53.1)
Mixed	47 (41.6)
Black	6 (5.3)
**Hypertension**, N (%)	34 (30.1)
**Diabetes Mellitus**, N (%)	5 (4.4)
**Deceased donor**, N (%)	91 (80.5)
**KDPI**, %	56 (35-82)
**KDRI**	1.06 (0.86-1.41)
**Donor Creatinine**, mg/dL (IQR)	1.40 (0.90-2.50)
**ECD**, N (%)	36 (39.6)
**CIT**, hours (IQR)	22.7 (18.8-29.0)

N: number; IQR: interquartile range; KDPI: Kidney Donor Profile Index; KDRI: Kidney Donor Risk Index; ECD: Expanded Criteria Donor; CIT: Cold Ischemia Time.

Table S2. Mean anti-SARS-CoV-2 IgG antibody titers by group and study visit.

	**Study visit**										**p**		
	**Screening**	**M1**	**M3**	**M6**	**M12**	**N_{scr}**	**N_{M1}**	**N_{M3}**	**N_{M6}**	**N_{M12}**	**Group**	**Time**	**Group and Time**
**IgG Titers, AU/mL**											0.003	0.006	0.005
Transplant	14,554.2±13,972.9	10,809.3±12,621.7	12,215.5±12,885.8	12,540.4±13,010.7	12,369.1±13,189.9	107	102	101	101	97			
Dialysis	13,164.0±15,174.7	15,267.8±16,096.2	15,016.2±15,346.1	18,503.5±14,581.0	12,818.2±12,171.6	102	95	93	91	82			
**IgG Titers, BAU/mL**											0.003	0.006	0.005
Transplant	2,065.3±1,984.1	1,534.9±1,792.3	1,734.6±1,829.8	1,780.7±1,847.5	1,756.4±1,873.0	107	102	101	101	97			
Dialysis	1,869.3±2,154,8	2,168.0±2,285.7	2,132.3±2,179.1	2,627.5±2,070.5	1,820.2±1,728.4	102	95	93	91	82			

Mean titers are represented in AU/mL and BAU/mL. p – descriptive level of the linear model with random effects. Time effect – Transplant: p=0.148; Dialysis: p=0.005.

Table S3. Primary vaccine schedule.

	**Total N = 221**	**Transplant N = 113**	**Dialysis N = 108**	**p**
**Vaccine,** N (%)				<0.001
ChAdOx1 nCoV-19	112 (50.7)	90 (79.6)	22 (20.4)	
CoronaVac	94 (42.5)	13 (11.5)	81 (75.0)	
BNT162b2	10 (4.5)	8 (7.1)	2 (1.9)	
Ad26.COV2.S	2 (0.9)	1 (0.9)	1 (0.9)	
Heterologous (BNT162b2 + ChAdOx1)	3 (1.4)	1 (0.9)	2 (1.9)	

N: Number. The heterologous schedule involved applying the first dose of the BNT162b2 vaccine, followed by the dose of ChAdOx1, with an interval of approximately three months between doses. The National Immunization Program did not foresee this scheme, which resulted in a failure at the time of immunization.

Table S4. Vaccine status of study population at screening.

Screening	**Total N = 221**	**Transplant N = 113**	**Dialysis N = 108**	**p**
***Doses received**, N (%)*				0.004
1	1 (0.5)	0 (0.0)	1 (0.9)	
2	39 (17.6)	27 (23.9)	12 (11.1)	
3	151 (68.3)	78 (69.0)	73 (67.6)	
4	30 (13.6)	8 (7.1)	22 (20.4)	
***Last dose received**, N (%)*				<0.001
BNT162b2	105 (47.5)	67 (59.3)	38 (35.2)	
CoronaVac	80 (36.2)	23 (20.4)	57 (52.8)	
ChAdOx1 nCoV-19	28 (12.7)	21 (18.6)	7 (6.5)	
Ad26.COV2.S	8 (3.6)	2 (1.8)	6 (5.6)	
**Time since last dose**, days (IQR)	106 (49-161)	90 (56-133)	161 (33-182)	0.005

N: number; IQR: interquartil range.

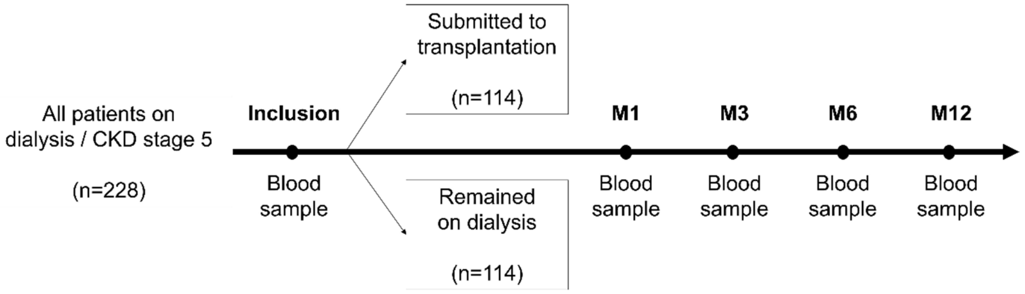

Figure S1. Flowchart of study visits.
